# Walkbot-Based Robot-Assisted Gait Training and Phase-Specific Lower-Limb Torque in Parkinson’s Disease: A Retrospective Comparative Study

**DOI:** 10.3390/brainsci16090989

**Published:** 2026-09-18

**Authors:** Gokhan Ozkocak, Rocco Salvatore Calabrò

**Affiliations:** 1 Department of Physical Medicine and Rehabilitation, Faculty of Medicine, Istanbul Aydin University, 34295 Istanbul, Turkey; 2 Spinal Cord Unit, IRCCS (Istituto di Ricovero e Cura a Carattere Scientifico) Centro Neurolesi “Bonino-Pulejo”, 98124 Messina, Italy

**Keywords:** Parkinson’s disease, neurorehabilitation, robot-assisted gait training, gait biomechanics, freezing of gait, phase-specific torque

## Abstract

**Highlights:**

**What are the main findings?**

- Walkbot-based robot-assisted gait training (RAGT) was associated with more favorable baseline-adjusted post-treatment outcomes than conventional rehabilitation in ambulatory individuals with mild-to-moderate Parkinson’s disease. Walkbot-derived phase-specific lower-limb torque was higher after RAGT during both stance and swing in the clinically more affected and contralateral limbs, with the largest adjusted between-group differences observed during stance.
- RAGT was also associated with better functional balance and lower self-reported freezing-related gait impairment, whereas disease-specific quality of life did not differ significantly between groups. These findings were largely maintained after additional adjustment for measured demographic and clinical characteristics.

**What are the implications of the main findings?**

- These findings suggest that Walkbot-derived phase-specific lower-limb torque may provide complementary quantitative information on locomotor changes associated with robotic gait rehabilitation that is not captured by clinical outcomes alone. Integrating device-derived kinetic measures with established clinical assessments may therefore provide a more comprehensive evaluation of rehabilitation response in Parkinson’s disease.
- The findings also support further prospective investigation of phase-specific lower-limb torque as an objective biomechanical outcome measure and its relationship with clinically relevant changes in balance and gait function.

**Abstract:**

Gait impairment, postural instability, and freezing of gait are major contributors to mobility limitations in Parkinson’s disease (PD). Robot-assisted gait training (RAGT) has emerged as a promising rehabilitation strategy; however, its association with phase-specific lower-limb kinetic outcomes remains insufficiently characterized. This study compared Walkbot-based RAGT and conventional rehabilitation in terms of Walkbot-derived phase-specific lower-limb torque, functional balance, self-reported freezing-related gait impairment, and health-related quality of life in individuals with PD. This retrospective comparative study included 60 individuals with idiopathic PD (Hoehn and Yahr stages II–III) who received either Walkbot-based RAGT (n = 30) or conventional rehabilitation (n = 30). Both groups completed 30 supervised sessions over 6 weeks. Biomechanical outcomes comprised Walkbot-derived lower-limb torque during the stance and swing phases for the clinically more affected and contralateral limbs. Clinical outcomes included the Berg Balance Scale (BBS), Freezing of Gait Questionnaire (FOG-Q), and Parkinson’s Disease Questionnaire-39 (PDQ-39). Between-group post-treatment differences were evaluated using analysis of covariance (ANCOVA), adjusting each outcome for its corresponding baseline value. After baseline adjustment, post-treatment phase-specific lower-limb torque was higher in the RAGT group for the more affected limb during swing (adjusted difference, 0.057 Nm/kg; 95% CI, 0.019–0.096; *p* = 0.004) and stance (0.145 Nm/kg; 95% CI, 0.098–0.193; *p* < 0.001), and for the contralateral limb during swing (0.051 Nm/kg; 95% CI, 0.006–0.096; *p* = 0.027) and stance (0.205 Nm/kg; 95% CI, 0.088–0.321; *p* = 0.001). The RAGT group also had higher baseline-adjusted BBS scores (adjusted difference, 5.78 points; 95% CI, 3.86–7.69; *p* < 0.001) and lower FOG-Q scores (−0.94 points; 95% CI, −1.27 to −0.61; *p* < 0.001). No significant between-group difference was observed for PDQ-39 (0.77 points; 95% CI, −0.16 to 1.69; *p* = 0.102). Walkbot-based RAGT was associated with more favorable baseline-adjusted phase-specific lower-limb torque, functional balance, and self-reported freezing-related gait impairment than conventional rehabilitation, whereas disease-specific quality of life did not differ significantly between groups. These findings highlight the potential value of integrating Walkbot-derived phase-specific kinetic assessment with clinical outcomes to provide a more quantitative characterization of rehabilitation-related locomotor changes in PD. Prospective randomized studies are needed to confirm these associations and establish the clinical utility of Walkbot-derived phase-specific lower-limb torque as an outcome measure.

## 1. Introduction

Parkinson’s disease (PD) is the second most common neurodegenerative disorder and is characterized by progressive dopaminergic neurodegeneration alongside dysfunction across multiple neural systems [1]. As the disease progresses, motor and non-motor symptoms increasingly affect functional independence and health-related quality of life. Gait dysfunction and postural instability are major contributors to disability, falls, hospitalization, and long-term dependence [2,3]. Although pharmacological treatment improves many motor symptoms, gait and balance problems often respond less completely to dopaminergic therapy and may become more prominent with disease progression. Rehabilitation therefore remains an important part of long-term PD management.

Gait dysfunction in PD involves more than a reduction in walking speed. Shorter steps, greater gait variability, longer double-support time, impaired postural control, and loss of walking automaticity are commonly observed [4,5,6]. Freezing of gait (FOG) may emerge as the disease progresses and can further restrict mobility. FOG is characterized by brief episodes in which effective forward progression is markedly reduced or absent despite the intention to walk. Its underlying mechanisms are complex, its response to medication is often incomplete, and its presence is strongly associated with falls and mobility limitations [7,8]. For these reasons, improving gait, balance, and functional mobility remains a central goal of rehabilitation in PD.

Exercise-based rehabilitation is recommended throughout the course of the disease. Conventional physiotherapy combining gait and balance exercises, external cueing, task-specific practice, and amplitude-based training can improve mobility and functional performance [9]. Lee Silverman Voice Treatment (LSVT) BIG is one example, using intensive, high-amplitude and repetitive movements to address the reduced movement amplitude characteristic of PD. However, responses to exercise vary, and it can be difficult to deliver a large number of reproducible gait cycles while simultaneously controlling loading, assistance, and gait parameters through therapist-assisted training alone [10].

Robot-assisted gait training (RAGT) offers a way to deliver intensive, repetitive stepping under more standardized conditions, with body-weight support, gait parameters, and robotic assistance adjusted according to the patient’s performance [11,12]. The Walkbot-G is a body-weight-supported, treadmill-based exoskeletal system with bilateral active actuation at the hip, knee, and ankle joints and integrated sensors for monitoring gait-related biomechanical outputs [13]. Studies of RAGT in PD have reported improvements in gait speed, step length, walking endurance, balance, mobility, and motor performance [14]. At the same time, systematic reviews point to considerable heterogeneity in training protocols, relatively small study populations, and low certainty of evidence for several outcomes [15]. Better quantitative characterization of the changes that accompany robotic gait rehabilitation is therefore still needed.

Most rehabilitation studies in PD have relied on clinical scales and spatiotemporal or kinematic gait measures. Kinetic changes have received considerably less attention. Bonacina et al. highlighted both the limited evidence on gait kinetics in PD and the heterogeneity of the available findings, identifying kinetic and propulsion-related measures as areas requiring further study [16]. Hayworth et al. also demonstrated altered lower-limb kinetics in mild-to-moderate PD, including reduced hip extension impulse during walking, while showing that participants could increase hip extension torque when locomotor demand was increased [17]. This suggests that phase-specific kinetic assessment may reveal aspects of walking performance that are not evident from clinical scales or spatiotemporal measures alone. Robotic systems provide an opportunity to examine such outputs during repeated gait cycles, although device-derived torque should be understood within the measurement framework of the robotic system rather than equated with joint-specific net moments obtained from conventional three-dimensional inverse-dynamics gait analysis.

Against this background, we examined whether Walkbot-based RAGT, compared with conventional rehabilitation, was associated with differences in Walkbot-derived phase-specific lower-limb torque during stance and swing, functional balance, self-reported freezing-related gait impairment, and health-related quality of life in ambulatory individuals with mild-to-moderate PD. By examining device-derived kinetic measures alongside established clinical outcomes, we aimed to characterize locomotor changes associated with robotic gait rehabilitation from both biomechanical and clinical perspectives. We hypothesized that RAGT would be associated with more favorable post-treatment phase-specific lower-limb torque and functional outcomes after accounting for baseline outcome values.

## 2. Materials and Methods

This study was designed as a retrospective, non-randomized comparative clinical study using routinely collected clinical and biomechanical assessment data from individuals with Parkinson’s disease who completed a standardized rehabilitation program at Medipol Acıbadem District Hospital’s neurological rehabilitation unit. The study was conducted and reported in accordance with the Strengthening the Reporting of Observational Studies in Epidemiology (STROBE) statement.

The investigators did not prospectively assign participants to treatment groups for research purposes. Instead, participants were retrospectively classified according to the rehabilitation modality they had received as part of routine clinical care.

All included participants had been considered clinically appropriate candidates for robot-assisted gait training by the treating rehabilitation team. However, robot-assisted rehabilitation was not routinely accessible to all eligible patients because of insurance coverage, financial limitations, and other access-related constraints. Consequently, treatment allocation reflected treatment accessibility within routine clinical practice rather than disease severity, expected treatment response, or investigator preference. Participants who received Walkbot-assisted gait training were classified into the robot-assisted gait training (RAGT) group, whereas those who received conventional physiotherapy because robotic rehabilitation was not accessible were classified into the conventional rehabilitation group.

The first 30 consecutive eligible participants in each treatment pathway who completed the predefined six-week rehabilitation program between September 2025 and May 2026 and had complete baseline and post-treatment assessments were included in the retrospective analysis. Participants were not selected according to their treatment response or post-treatment outcomes. Nevertheless, because treatment allocation was not randomized, the possibility of residual selection bias and unmeasured confounding cannot be excluded.

The study protocol was approved by the University Ethics Committee (Approval No: E-10840098-202.3.02-5564) on 22 August 2025. Written informed consent was obtained from all participants before rehabilitation. All study procedures were conducted in accordance with the ethical principles of the Declaration of Helsinki.

### 2.1. Participants

A total of 60 individuals with idiopathic Parkinson’s disease were included in the study, comprising 30 participants in the RAGT group and 30 participants in the conventional physiotherapy group. The diagnosis of Parkinson’s disease had been established by a neurologist according to the Movement Disorder Society (MDS) clinical diagnostic criteria.

Participants were included if they met the following criteria:

- Diagnosis of idiopathic Parkinson’s disease;
- Hoehn and Yahr stage II–III;
- Ability to ambulate independently, with or without an assistive device;
- Stable dopaminergic medication regimen before study entry, with no changes in antiparkinsonian medication throughout the six-week rehabilitation program

Exclusion criteria included:

- Severe musculoskeletal disorders affecting gait performance;
- Significant cognitive impairment that could interfere with participation in rehabilitation;
- Other neurological or orthopedic disorders likely to influence gait or functional mobility.

Baseline demographic and clinical characteristics were extracted from the medical records. In addition to age and sex, these included disease duration, Hoehn and Yahr stage, antiparkinsonian medication regimen, levodopa equivalent daily dose (LEDD), and the use of an assistive mobility device for ambulation. Hoehn and Yahr stage II and III distributions were recorded separately for each treatment group. Assistive-device use was categorized according to baseline ambulatory status, with the type of device additionally recorded when applicable.

Total LEDD was calculated from the medication regimen recorded at baseline using established levodopa-equivalent conversion factors [18]. Disease duration and Hoehn and Yahr stage were used to characterize disease chronicity and clinical severity, respectively, whereas LEDD was used to quantify overall antiparkinsonian medication burden rather than as a surrogate measure of disease progression.

All clinical and biomechanical assessments were performed during the ON medication state to minimize variability related to dopaminergic medication timing.

### 2.2. Walkbot-G System Architecture and Robotic Assistance

**Figure 1:**
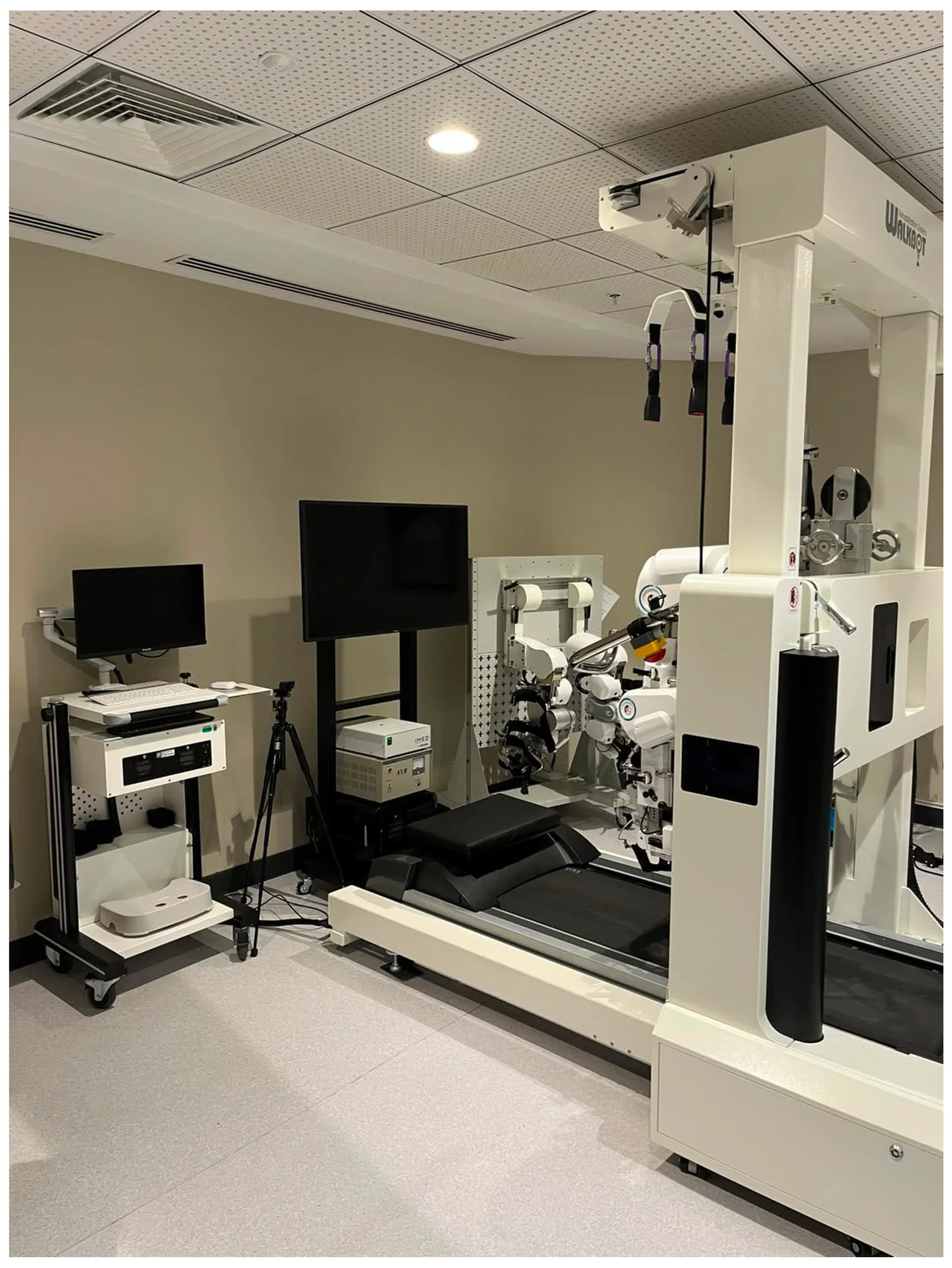
Walkbot-G system (P&S Mechanics, Seoul, Republic of Korea), a body-weight-supported treadmill.

Robot-assisted gait training was performed using the Walkbot-G system (P&S Mechanics, Seoul, Republic of Korea), a body-weight-supported treadmill-based robotic gait-training system incorporating a bilateral lower-limb exoskeleton (Figure 1). The system provides coordinated actuation of the hip, knee, and ankle joints of both lower extremities, guiding lower-limb movement predominantly in the sagittal plane during treadmill walking. The exoskeleton is secured to the participant using adjustable lower-limb interfaces and operates in combination with a harness-based body-weight-support system. The Walkbot platform has previously been described as a reliable system for standardized robotic gait training and biomechanical assessment [13].

Before training and biomechanical assessment, the system was individually configured according to each participant’s anthropometric characteristics. Exoskeletal segment lengths and joint alignment were adjusted to approximate the anatomical hip, knee, and ankle axes. Body-weight support, walking speed, step length, and robotic assistance were subsequently individualized according to gait performance, postural stability, fatigue, active participation, and clinical tolerance.

The Walkbot-G provides active motorized assistance at the bilateral hip, knee, and ankle joints. For the present retrospective analysis, the available kinetic variable consisted of device-derived lower-limb torque outputs synchronized with the stance and swing phases of gait. These values were therefore analyzed as Walkbot-derived phase-specific lower-limb torque rather than as isolated joint-specific hip, knee, or ankle net moments.

Accordingly, the reported values should be interpreted as device-derived kinetic outputs recorded during robot-assisted walking and not as conventional joint-specific net moments obtained using three-dimensional inverse-dynamics gait analysis. Robotic assistance was delivered within an impedance-based patient–robot interaction framework, allowing the degree of robotic guidance and resistance to be adjusted according to participant performance and therapeutic goals. Training progression was implemented through Passive, Active, and Resistive modes, as described below. Because the detailed internal control algorithms and threshold values are proprietary, no assumptions regarding their specific mathematical implementation were made in the present analysis.

### 2.3. Rehabilitation Protocols

All participants underwent a 6-week rehabilitation program consisting of 30 supervised sessions (five sessions per week), with each session lasting 60 min. Participants were retrospectively classified according to the rehabilitation modality received as part of routine clinical care: Walkbot-assisted robot-assisted gait training (RAGT) or conventional rehabilitation. The two rehabilitation pathways were therefore matched in treatment frequency, session duration, and overall rehabilitation period, while differing in the mode and content of training.

#### 2.3.1. Walkbot-Assisted Robot-Assisted Gait Training

Participants in the RAGT group completed 30 Walkbot-assisted gait-training sessions over six weeks (five sessions per week; 60 min per session). Each session targeted approximately 1000 steps, and the gait cycle duration was set at 3.6 s per two steps. Training followed a standardized three-stage progression consisting of Passive, Active, and Resistive modes, designed to progressively transition participants from adaptation to the robotic gait pattern toward greater voluntary participation and muscular demand.

During the first five sessions (sessions 1–5), training was performed in Passive mode to facilitate familiarization with the exoskeleton and adaptation to the predefined gait trajectory. The subsequent 15 sessions (sessions 6–20) were performed in Active mode, with greater emphasis on voluntary participation and patient-robot interaction. Participants were encouraged to actively contribute to the gait cycle rather than relying predominantly on robotic guidance. The final 10 sessions (sessions 21–30) were performed in Resistive mode to progressively increase muscular demand during walking. In this final stage, resistance was introduced against lower-limb movement while the programmed gait pattern was maintained, requiring greater voluntary effort during the gait cycle.

Body-weight support was initially set at approximately 30–40% and was progressively reduced as tolerated. Walking speed, step length, body-weight support, and robotic assistance were individually adjusted according to gait performance, postural stability, fatigue, and the participant’s ability to actively contribute to stepping. Thus, although all participants followed the same predefined Passive-Active-Resistive progression, specific training parameters were individualized according to clinical performance and tolerance. All sessions were supervised by physiotherapists experienced and certified in neurological robotic rehabilitation.

#### 2.3.2. Conventional Rehabilitation

Participants in the LSVT BIG-based rehabilitation group completed 30 therapist-supervised rehabilitation sessions over six weeks (five sessions per week; 60 min per session). The rehabilitation program was based on LSVT BIG principles and emphasized high-amplitude, repetitive, and task-specific movements aimed at addressing impaired movement scaling in Parkinson’s disease. Training incorporated large-amplitude whole-body movements, functional component tasks, gait-related activities, dynamic postural control, functional mobility, lower-limb strengthening, and repeated practice of movement strategies intended to facilitate transfer to everyday motor activities [10]. Exercise complexity and task difficulty were progressively individualized according to each participant’s functional performance, postural stability, and tolerance while maintaining the amplitude-oriented principles of LSVT BIG throughout the intervention period.

Both rehabilitation groups therefore received the same treatment frequency and duration (30 supervised 60-min sessions over six weeks). The principal difference between the rehabilitation pathways was the mode of gait-oriented training: the RAGT group received exoskeletal gait training with a predefined Passive–Active–Resistive progression, whereas the LSVT BIG-based rehabilitation group received therapist-supervised amplitude-oriented and task-specific exercise without robotic assistance.

### 2.4. Sensor-Based Biomechanical Data Acquisition and Phase-Specific Lower-Limb

Biomechanical data were obtained directly from the integrated sensing and gait-analysis system of the Walkbot-G. During robot-assisted walking, the system continuously acquired lower-limb biomechanical signals across successive gait cycles and synchronized these data with device-generated gait-phase information. The gait cycle was classified by the embedded system into stance and swing phases, enabling kinetic output to be evaluated according to the functional phase of gait. The present retrospective analysis used the stance and swing phase classifications provided by the Walkbot system.

Biomechanical assessments were performed at baseline and after completion of the six-week rehabilitation program under standardized conditions. Participants in both groups underwent the same Walkbot-based gait-analysis assessment during the ON medication state. The same standardized Walkbot-based assessment protocol was applied to both groups in the ON-medication state. For each participant, biomechanical data were collected over 250 gait cycles during both the pre- and post-intervention assessments. Walkbot-derived torque data were classified according to the stance and swing phases and summarized across the recorded gait cycles for subsequent analysis. Before each assessment, the exoskeleton was individually fitted according to the participant’s anthropometric characteristics, including adjustment of segment lengths and alignment of the robotic joint axes with the corresponding anatomical axes. The same standardized gait-analysis protocol and device settings were used for baseline and post-rehabilitation assessments.

The primary biomechanical outcome was Walkbot-derived phase-specific lower-limb torque. Torque output was quantified separately during the stance and swing phases for the clinically more affected and contralateral limbs. The clinically more affected side was defined as the side exhibiting predominant parkinsonian motor manifestations at baseline, and the same side designation was maintained for all baseline and post-rehabilitation comparisons.

Torque values were normalized to body mass and expressed as Nm/kg to facilitate between-participant comparison. Accordingly, four phase- and side-specific biomechanical outcomes were evaluated: stance- and swing-phase lower-limb torque for the clinically more affected limb and stance- and swing-phase lower-limb torque for the contralateral limb.

Although the Walkbot-G provides active motorized actuation at the bilateral hip, knee, and ankle joints and permits separate sensor-derived torque measurement at the hip and knee, the primary torque variable analyzed in the present study was the device-derived phase-specific lower-limb torque output rather than an isolated joint-specific net moment. An equivalent joint-specific ankle torque output was not available and was therefore not analyzed. The reported torque values should consequently be interpreted as Walkbot-derived kinetic outputs recorded during robot-assisted walking and not as conventional hip-, knee-, or ankle-joint net moments obtained using three-dimensional inverse-dynamics gait analysis.

### 2.5. Clinical Assessment

Clinical outcomes were assessed at baseline and after completion of the six-week rehabilitation program. All assessments were performed during the ON medication state under standardized clinical conditions.

Functional balance was assessed using the Berg Balance Scale (BBS), a 14-item performance-based measure of static and dynamic balance during functional activities, including transfers, standing, reaching, turning, and stepping. Each item is scored from 0 to 4, yielding a total score ranging from 0 to 56, with higher scores indicating better functional balance.

Freezing of gait (FOG) is characterized by brief, episodic absence or marked reduction of effective forward progression of the feet despite the intention to walk. Freezing-related gait impairment was assessed using the Freezing of Gait Questionnaire (FOG-Q), a six-item patient-reported questionnaire evaluating freezing episodes and their impact on walking and daily mobility. Each item is scored from 0 to 4, yielding a total score ranging from 0 to 24, with higher scores indicating greater freezing-related impairment. Because the FOG-Q is a patient-reported measure rather than an instrumented assessment of freezing episodes, changes in FOG-Q scores were interpreted as changes in self-reported freezing-related gait impairment and not as direct quantitative measures of freezing biomechanics.

Health-related quality of life was assessed using the 39-item Parkinson’s Disease Questionnaire (PDQ-39), a disease-specific patient-reported outcome measure evaluating the impact of Parkinson’s disease across multiple domains of daily functioning and well-being. Higher scores indicate poorer health-related quality of life.

### 2.6. Statistical Analysis

Statistical analyses were performed using IBM SPSS Statistics version 26.0 (IBM Corp., Armonk, NY, USA). Continuous variables were summarized as mean ± standard deviation (SD) or median and interquartile range (IQR), as appropriate, whereas categorical variables were presented as frequencies and percentages. The distribution of continuous variables was assessed using the Kolmogorov–Smirnov test together with visual inspection of the data distribution.

Baseline demographic and clinical characteristics were compared between the RAGT and conventional rehabilitation groups using the independent-samples *t*-test for normally distributed continuous variables and the Mann–Whitney U test for non-normally distributed or ordinal variables. Categorical variables were compared using the chi-square test or Fisher’s exact test, as appropriate. Hoehn and Yahr stage was treated as an ordinal variable and compared between groups using the Mann–Whitney U test.

For the primary between-group analyses, separate analysis of covariance (ANCOVA) models were performed for each outcome. The post-treatment value was entered as the dependent variable, treatment group (RAGT vs. conventional rehabilitation) as the fixed factor, and the corresponding baseline value of the outcome as a covariate. This approach was applied to the four Walkbot-derived phase-specific lower-limb torque outcomes—stance- and swing-phase torque for the clinically more affected and contralateral limbs—as well as to the Berg Balance Scale (BBS), Freezing of Gait Questionnaire (FOG-Q), and 39-item Parkinson’s Disease Questionnaire (PDQ-39). Adjusted between-group differences were estimated as RAGT minus conventional rehabilitation and are reported with 95% confidence intervals (CIs).

To evaluate the robustness of the primary findings to measured baseline confounding, sensitivity analyses were additionally performed using multivariable ANCOVA models. These models included the corresponding baseline outcome, age, sex, education, disease duration, LEDD, and Hoehn and Yahr stage, in addition to treatment group. Given the sample size, these expanded models were considered sensitivity analyses rather than the primary inferential models.

All statistical tests were two-sided, and a *p*-value < 0.05 was considered statistically significant.

## 3. Results

### 3.1. Participant Characteristics

Sixty participants were included in the retrospective analysis. Based on the rehabilitation modality received during routine clinical care, 30 participants were classified in the robot-assisted gait training (RAGT) group and 30 in the conventional rehabilitation group.

Baseline demographic and clinical characteristics are presented in Table 1. No statistically significant between-group differences were observed in age, sex, education, disease duration, levodopa equivalent daily dose (LEDD), or Hoehn and Yahr stage (all *p* > 0.05). The distributions of Hoehn and Yahr stages 2, 2.5, and 3 were 33.3%, 36.7%, and 30.0%, respectively, in the RAGT group and 30.0%, 40.0%, and 30.0% in the conventional rehabilitation group (overall between-group comparison, *p* = 0.869). Assistive mobility device use was infrequent in both groups, occurring in 3 participants (10.0%) in the RAGT group and 2 participants (6.7%) in the conventional rehabilitation group.

### 3.2. Biomechanical Outcomes

Baseline and post-treatment Walkbot-derived phase-specific lower-limb torque values are presented in Table 2. In the primary ANCOVA analyses, post-treatment torque values were higher in the RAGT group across all four phase- and side-specific outcomes after adjustment for the corresponding baseline torque value.

For the clinically more affected limb, the observed mean swing-phase torque increased from 0.260 ± 0.117 Nm/kg at baseline to 0.424 ± 0.109 Nm/kg after treatment in the RAGT group and from 0.268 ± 0.082 to 0.371 ± 0.095 Nm/kg in the conventional rehabilitation group. After adjustment for baseline swing-phase torque, the adjusted between-group difference at post-treatment was 0.057 Nm/kg (95% CI, 0.019 to 0.096; *p* = 0.004).

During stance, the observed mean torque of the clinically more affected limb increased from 0.205 ± 0.131 to 0.460 ± 0.176 Nm/kg in the RAGT group and from 0.224 ± 0.120 to 0.332 ± 0.121 Nm/kg in the conventional rehabilitation group. The baseline-adjusted between-group difference was 0.145 Nm/kg (95% CI, 0.098 to 0.193; *p* < 0.001).

For the contralateral limb, mean swing-phase torque increased from 0.349 ± 0.132 to 0.473 ± 0.156 Nm/kg in the RAGT group and from 0.322 ± 0.133 to 0.399 ± 0.121 Nm/kg in the conventional rehabilitation group. The adjusted between-group difference was 0.051 Nm/kg (95% CI, 0.006 to 0.096; *p* = 0.027). During stance, mean torque increased from 0.339 ± 0.178 to 0.653 ± 0.318 Nm/kg in the RAGT group and from 0.300 ± 0.137 to 0.421 ± 0.145 Nm/kg in the conventional rehabilitation group, corresponding to an adjusted between-group difference of 0.205 Nm/kg (95% CI, 0.088 to 0.321; *p* = 0.001) (Table 2).

### 3.3. Clinical Outcomes

Baseline and post-treatment clinical outcomes and baseline-adjusted between-group comparisons are presented in Table 2.

For functional balance, the observed mean BBS score increased from 24.27 ± 7.44 to 37.53 ± 7.43 in the RAGT group and from 25.40 ± 7.14 to 32.80 ± 7.85 in the conventional rehabilitation group. After adjustment for baseline BBS, the post-treatment score was higher in the RAGT group, with an adjusted between-group difference of 5.78 points (95% CI, 3.86 to 7.69; *p* < 0.001).

FOG-Q scores decreased from 10.23 ± 1.85 to 8.23 ± 1.59 in the RAGT group and from 10.53 ± 1.63 to 9.43 ± 1.68 in the conventional rehabilitation group. After adjustment for baseline FOG-Q, the RAGT group had a lower post-treatment score, with an adjusted between-group difference of −0.94 points (95% CI, −1.27 to −0.61; *p* < 0.001). This finding indicates lower self-reported freezing-related gait impairment at post-treatment in the RAGT group.

PDQ-39 scores decreased from 56.93 ± 12.78 to 52.21 ± 12.13 in the RAGT group and from 56.89 ± 12.48 to 51.40 ± 12.10 in the conventional rehabilitation group. After adjustment for baseline PDQ-39, the between-group difference at post-treatment was 0.77 points (95% CI, −0.16 to 1.69; *p* = 0.102) and was not statistically significant (Table 2).

### 3.4. Sensitivity Analyses

Sensitivity analyses were performed to examine the robustness of the primary findings to additional adjustment for measured baseline characteristics. In addition to the corresponding baseline outcome, the expanded ANCOVA models included age, sex, education, disease duration, LEDD, and Hoehn and Yahr stage as covariates. Overall, the direction and magnitude of the estimated between-group differences were largely consistent with those obtained in the primary baseline-adjusted models (Table 3).

For the Walkbot-derived biomechanical outcomes, the multivariable-adjusted between-group differences remained statistically significant for all four phase- and side-specific torque measures. For the clinically more affected limb, the adjusted difference was 0.062 Nm/kg (95% CI, 0.022 to 0.102; *p* = 0.003) during swing and 0.147 Nm/kg (95% CI, 0.097 to 0.197; *p* < 0.001) during stance. For the contralateral limb, the corresponding adjusted differences were 0.047 Nm/kg (95% CI, 0.001 to 0.092; *p* = 0.047) during swing and 0.209 Nm/kg (95% CI, 0.084 to 0.334; *p* = 0.002) during stance.

The clinical findings were similarly maintained after multivariable adjustment. The adjusted between-group difference was 5.58 points (95% CI, 3.58 to 7.58; *p* < 0.001) for BBS and −0.96 points (95% CI, −1.31 to −0.61; *p* < 0.001) for FOG-Q. In contrast, the adjusted between-group difference in PDQ-39 remained non-significant (0.80 points; 95% CI, −0.17 to 1.78; *p* = 0.105) (Table 3).

## 4. Discussion

This retrospective comparative study showed that Walkbot-based robot-assisted gait training (RAGT) was associated with more favorable baseline-adjusted post-treatment outcomes than conventional rehabilitation across all four Walkbot-derived phase-specific lower-limb torque measures and functional balance in ambulatory individuals with mild-to-moderate Parkinson’s disease. The adjusted between-group differences were particularly evident during the stance phase, both for the clinically more affected and contralateral limbs. Importantly, these findings remained largely consistent in sensitivity analyses additionally adjusting for age, sex, education, disease duration, levodopa equivalent daily dose (LEDD), and Hoehn and Yahr stage. Taken together, these results suggest that RAGT may be associated with favorable changes in phase-specific lower-limb kinetic performance and selected clinical measures beyond those observed with conventional rehabilitation.

The distinction between stance and swing is important when considering gait dysfunction in Parkinson’s disease. Most gait studies in PD have focused on spatiotemporal and kinematic abnormalities, while kinetic characteristics have received considerably less attention. A recent systematic review and meta-analysis showed that people with PD walk with shorter strides, lower walking speed and swing time, longer double-support time, and reduced sagittal-plane excursion at the hip, knee, and ankle. Kinetic abnormalities have also been reported, although the available evidence is smaller and more heterogeneous [17]. Phase-specific analysis may therefore add information that is not captured by a single overall measure of gait performance.

Stance places substantial demands on lower-limb support and control. The limb must accept body weight, support the body as the center of mass progresses forward, and contribute to progression toward the next step. These functions are relevant to the characteristic gait changes seen in PD, including longer double-support periods and altered lower-limb kinetic and propulsive characteristics [4,6,16]. Hayworth et al. found reduced hip extension impulse during walking in people with mild-to-moderate PD, but also showed that hip extension torque could increase when locomotor demands were raised. This suggests that the capacity to adapt kinetic output may not be completely lost in this population [17].

In our study, the adjusted between-group differences were numerically larger during stance than during swing on both the clinically more affected side (0.145 vs. 0.057 Nm/kg) and the contralateral side (0.205 vs. 0.051 Nm/kg). We did not directly test the difference between the stance and swing effects, so this pattern should not be taken to indicate a preferential treatment effect on stance. It does, however, show that the separation between groups was most evident while the limb was weight-bearing. Higher stance-phase torque after RAGT may reflect a greater capacity to generate lower-limb kinetic output during support and progression.

Swing-phase torque addresses a different part of the walking cycle. Effective limb advancement requires sufficient shortening of the limb, foot clearance, and appropriate positioning for the next initial contact. In PD, shorter swing time and stride length and reduced sagittal-plane lower-limb excursion may interfere with this process [17]. Altered foot-clearance characteristics have also been reported, including in people with relatively early disease [19]. The higher swing-phase torque observed after RAGT may thus reflect greater kinetic output during limb advancement. Interestingly, the same pattern was seen on the contralateral side as well as the clinically more affected side. The response was therefore not limited to the limb with predominant Parkinsonian motor manifestations. Whether this was accompanied by longer steps, better foot clearance, or faster overground walking cannot be answered from the present data because these variables were not systematically recorded.

Looking at stance and swing together provides a broader picture than either phase alone. Parkinsonian gait is not simply a problem of reduced movement amplitude or force generation. Scaling successive steps and coordinating the two lower limbs are also important components of gait dysfunction. Walking requires continuous alternation between weight-bearing and limb advancement, even when the clinical manifestations of PD are clearly asymmetric. In this study, higher post-treatment torque in the RAGT group was found during both phases and on both sides. This bilateral, phase-dependent pattern may therefore be useful when considering how robotic kinetic measures capture gait performance in PD. Impaired left–right stepping coordination has also been described in PD, particularly in relation to freezing of gait [20], although bilateral coordination itself was not measured in our study.

The training protocol offers a reasonable context for these findings. Robotic gait training repeatedly exposes the patient to alternating stance and swing within the task of walking itself. Walkbot-G provides bilateral hip, knee, and ankle actuation, while body-weight support, gait parameters, and robotic assistance can be modified according to individual performance. Training in this study progressed from Passive to Active and then Resistive modes, increasing the demand for active participation over the course of the program. Repeated practice under these changing conditions may allow patients to contribute more actively during different parts of the gait cycle. Task-specific repetition and sensorimotor feedback are well-established elements of motor learning in neurological rehabilitation [11,12]. At the same time, our data do not show why torque changed. The findings describe a phase-specific kinetic response associated with RAGT rather than a specific neuromuscular or neuroplastic mechanism.

The balance results add a clinical dimension to the biomechanical findings. After baseline adjustment, the post-treatment BBS score was 5.78 points higher in the RAGT group (95% CI, 3.86–7.69; *p* < 0.001). This is consistent with previous systematic reviews reporting improvements in balance and mobility-related outcomes after robot-assisted gait training in PD [14,21]. This between-group difference is notable given that the conventional rehabilitation program included LSVT BIG, an active amplitude-based intervention with established effects on motor function in PD. A recent systematic review and meta-analysis reported improvements in balance and gait following LSVT BIG, although effects were not consistent across all mobility and quality-of-life outcomes [22]. The larger numerical differences in stance-phase torque are interesting alongside the BBS result because lower-limb support and postural control are both required during stance. Still, the two findings cannot be assumed to have a direct causal relationship. BBS assesses a range of functional balance tasks and does not identify the biomechanical basis of improvement. Walkbot-G also predominantly guides lower-limb movement in the sagittal plane and does not provide the same frontal- and transverse-plane robotic degrees of freedom as systems with additional pelvic freedom. Although pelvic tilting is permitted during Walkbot training, pelvic kinematics were not measured here, so the balance findings cannot be attributed to this feature.

The FOG-Q findings deserve a similarly careful interpretation. Freezing occurs within a broader disturbance of locomotor control rather than as an isolated interruption of forward progression. Impaired step scaling, gait rhythmicity, bilateral coordination, and dynamic postural control have all been linked to FOG [20]. A systematic review of biomechanical changes preceding freezing episodes also identified shorter stride length, slower gait, longer double-support time, postural instability, and alterations in sagittal-plane lower-limb motion [23]. These observations make the organization of successive gait phases relevant when considering freezing in PD.

The presence of between-group torque differences during both stance and swing is therefore an interesting biomechanical finding alongside the reduction in self-reported freezing-related impairment. Repetitive rhythmic stepping and consistent sensorimotor input during robotic gait training may provide a structured setting for practicing the temporal organization of walking [7,24,25]. However, the two findings should remain separate in their interpretation. After baseline adjustment, the RAGT group had a 0.94-point lower FOG-Q score (95% CI, −1.27 to −0.61; *p* < 0.001), but FOG-Q is patient-reported and does not objectively quantify freezing episodes. We therefore cannot determine whether the torque differences were related to the change in freezing, nor whether RAGT altered the biomechanics of FOG. Studies combining phase-specific kinetic measures with wearable sensors or instrumented detection of freezing episodes would help clarify this relationship.

PDQ-39 showed a different pattern. Despite the differences in torque, balance, and FOG-Q, post-treatment PDQ-39 did not differ significantly between groups after baseline adjustment. This is perhaps not unexpected given the broader scope of the questionnaire. Quality of life in PD is shaped by motor symptoms as well as mood, cognition, fatigue, sleep, pain, and social participation, many of which may not change in parallel with gait performance during a six-week intervention [26]. The PDQ-39 result therefore indicates that the between-group differences observed in gait-related outcomes were not accompanied by a detectable difference in disease-specific quality of life over the same period.

Our results are broadly consistent with the growing literature on robot-assisted rehabilitation in PD. Meta-analyses have reported improvements in balance, gait performance, walking endurance, and motor function after robotic gait interventions, while also noting considerable heterogeneity in training protocols and outcome measures [15,16,19]. Objective kinetic measures remain relatively uncommon compared with clinical scales and spatiotemporal gait parameters [17]. The present study adds to this literature by examining Walkbot-derived lower-limb torque separately according to gait phase and clinical side alongside established clinical outcomes. We recently used a similar phase-specific approach in chronic hemiplegia [27]. Although the underlying motor disorders are clearly different, both studies illustrate how device-derived kinetic outputs may complement conventional clinical measures in neurological gait rehabilitation.

Several strengths and limitations should be considered. The study combines device-derived phase-specific biomechanical data with established clinical outcomes in a standardized single-center rehabilitation setting. Baseline-adjusted analyses were used for the primary comparisons, and the main estimates changed little after additional adjustment for age, sex, education, disease duration, LEDD, and Hoehn and Yahr stage. Nevertheless, this was a retrospective, non-randomized study, and causal conclusions cannot be drawn. Selection bias and residual confounding remain possible. Access to RAGT was partly influenced by financial considerations, while detailed socioeconomic information was not systematically available. Adjustment for measured characteristics cannot account for unmeasured differences between the groups. The moderate sample size and single-center design further limit generalizability.

Other limitations arise from the retrospective clinical dataset. Overground walking velocity was not systematically recorded, and long-term follow-up was unavailable. We also lacked electromyography, detailed pelvic kinematics, objective freezing detection, and neurophysiological or neuroimaging assessments. Falls and daily-life mobility were not directly evaluated. As a result, we cannot determine whether the phase-specific torque differences translated into better propulsion, foot clearance, interjoint coordination, fewer freezing episodes, or improved mobility outside the rehabilitation setting. Finally, the participants were ambulatory and had mild-to-moderate PD, and the findings may not apply to patients with advanced disease or substantial cognitive or mobility impairment.

Overall, Walkbot-based RAGT was associated with higher baseline-adjusted lower-limb torque during both stance and swing and on both the clinically more affected and contralateral sides after six weeks of rehabilitation. These biomechanical differences were accompanied by better functional balance and lower self-reported freezing-related impairment, while PDQ-39 did not differ between groups. The bilateral and phase-specific pattern suggests that examining kinetic output according to the functional demands of the gait cycle may provide information beyond a single overall measure of walking performance. Prospective randomized studies incorporating objective freezing assessment, and longer-term follow-up are needed to determine the clinical significance of these device-derived measures and whether the observed differences persist beyond the treatment period.

## 5. Conclusions

Walkbot-based robot-assisted gait training (RAGT) was associated with higher baseline-adjusted post-treatment Walkbot-derived phase-specific lower-limb torque and balance performance, as well as lower self-reported freezing-related gait impairment, compared with conventional rehabilitation in ambulatory individuals with mild-to-moderate Parkinson’s disease. These associations were largely maintained after additional adjustment for measured demographic and clinical characteristics, whereas no significant between-group difference was observed in disease-specific quality of life. By integrating phase-specific device-derived kinetic measures with established clinical outcomes, this study provides a more comprehensive evaluation of rehabilitation-related locomotor adaptations than approaches relying on clinical or spatiotemporal gait assessments alone. The findings highlight the potential value of objective biomechanical assessment for monitoring treatment response during robot-assisted gait rehabilitation and support RAGT as a valuable component of comprehensive neurorehabilitation for Parkinson’s disease.

## Figures and Tables

**Table 1:** Baseline Characteristics and Clinical Measures.

Variable	Robotic (n = 30)	Conventional (n = 30)	*p*-Value
**Age (years)** **Mean ± SD**	64.9 ± 9.7	65.6 ± 8.7	0.748 *
**Gender (Female/Male)** **Mean ± SD**	11/19	10/20	0.787 **
**Education (years)** **Mean ± SD**	13.86 ± 2.86	12.93 ± 2.91	0.216 *
**Disease duration (years)** **Mean ± SD**	6.4 ± 1.9	5.8 ± 1.5	0.249 *
**LEDD (mg/day)** **Mean ± SD**	432.56 ± 103.47	429.2 ± 94.2	0.896 *
**Hoehn and Yahr stage** **Mean ± SD**	2.48 ± 0.4	2.5 ± 0.4	0.869 ^†^
**Hoehn and Yahr stage** **2/2.5/3, n (%)**	10/11/9 (33.3%)/(36.7%)/(30%)	9/12/9 (30%)/(40%)/(30%)	
**BBS** **Median (IQR)**	23 (IQR: 17.8–29.5)	22.5 (IQR: 20.5–31.5)	0.604 ^†^
**PDQ-39** **Mean ± SD**	56.9 ± 12.8	56.9 ± 12.5	0.990 *
**FOG-Q** **Mean ± SD**	10.23 ± 1.85	10.53 ± 1.63	0.508 *

SD: Standard deviation; IQR: Inter quartile range; BBS: Berg Balance Scale; PDQ-39: Parkinson’s Disease Questionnaire-39; FOG-Q: Freezing of Gait Questionnaire; LEDD: levodopa equivalent daily dose. * Independent sample *t*-test. ** Chi-square test. ^†^ Mann-Whitney U test.

**Table 2:** Baseline and post-treatment biomechanical and clinical outcomes and baseline-adjusted between-group comparisons.

Outcome	RAGT Pre	RAGT Post	Conventional Pre	Conventional Post	Adjusted Between-Group Difference (95% CI)	ANCOVA *p*
**More affected limb—Swing torque (Nm/kg)**	0.260 ± 0.117	0.424 ± 0.109	0.268 ± 0.082	0.371 ± 0.095	0.057 (0.019 to 0.096)	0.004
**More affected limb—Stance torque (Nm/kg)**	0.205 ± 0.131	0.460 ± 0.176	0.224 ± 0.120	0.332 ± 0.121	0.145 (0.098 to 0.193)	<0.001
**Contralateral limb—Swing torque (Nm/kg)**	0.349 ± 0.132	0.473 ± 0.156	0.322 ± 0.133	0.399 ± 0.121	0.051 (0.006 to 0.096)	0.027
**Contralateral limb—Stance torque (Nm/kg)**	0.339 ± 0.178	0.653 ± 0.318	0.300 ± 0.137	0.421 ± 0.145	0.205 (0.088 to 0.321)	0.001
**BBS**	24.27 ± 7.44	37.53 ± 7.43	25.40 ± 7.14	32.80 ± 7.85	5.78 (3.86 to 7.69)	<0.001
**FOG-Q**	10.23 ± 1.85	8.23 ± 1.59	10.53 ± 1.63	9.43 ± 1.68	−0.94 (−1.27 to −0.61)	<0.001
**PDQ-39**	56.93 ± 12.78	52.21 ± 12.13	56.89 ± 12.48	51.40 ± 12.10	0.77 (−0.16 to 1.69)	0.102

ANCOVA: analysis of covariance; CI: confidence interval; BBS: Berg Balance Scale; FOG-Q: Freezing of Gait Questionnaire; PDQ-39: 39-item Parkinson’s Disease Questionnaire.

**Table 3:** Sensitivity Analysis of Between-Group Differences in Biomechanical and Clinical Outcomes After Multivariable Adjustment.

Outcome	Primary ANCOVA: Adjusted Difference (95% CI), *p*	Fully Adjusted Sensitivity Model: Adjusted Difference (95% CI), *p*
**More affected—Swing**	0.057 (0.019–0.096), *p* = 0.004	0.062 (0.022–0.102), *p* = 0.003
**More affected—Stance**	0.145 (0.098–0.193), *p* < 0.001	0.147 (0.097–0.197), *p* < 0.001
**Contralateral—Swing**	0.051 (0.006–0.096), *p* = 0.027	0.047 (0.001–0.092), *p* = 0.047
**Contralateral—Stance**	0.205 (0.088–0.321), *p* = 0.001	0.209 (0.084–0.334), *p* = 0.002
**BBS**	5.78 (3.86–7.69), *p* < 0.001	5.58 (3.58–7.58), *p* < 0.001
**FOG-Q**	−0.94 (−1.27 to −0.61), *p* < 0.001	−0.96 (−1.31 to −0.61), *p* < 0.001
**PDQ-39**	0.77 (−0.16–1.69), *p* = 0.102	0.80 (−0.17–1.78), *p* = 0.105

ANCOVA: analysis of covariance; CI: confidence interval; BBS: Berg Balance Scale; FOG-Q: Freezing of Gait Questionnaire; PDQ-39: 39-item Parkinson’s Disease Questionnaire.

## Data Availability

The data presented in this study are available on request from the corresponding author. (The data are not publicly available due to privacy or ethical restrictions).
